# Designing Competencies for Chronic Disease Practice

**Published:** 2010-02-15

**Authors:** Kathleen M. Quinlan, Amy Slonim, Fran C. Wheeler, Suzanne M Smith

**Affiliations:** Oxford Learning Institute. At the time of this study Dr Quinlan was affiliated with Concept Systems, Inc, Ithaca, New York; Michigan Public Health Institute, Okemos, Michigan. Dr Slonim is affiliated with the Centers for Disease Control and Prevention, Washington, DC; National Association of Chronic Disease Directors, Atlanta, Georgia; University of Massachusetts-Amherst, Amherst, Massachusetts

## Abstract

**Introduction:**

Competencies are the cornerstone of effective public health practice, and practice specialties require competencies specific to their work. Although more than 30 specialty competency sets have been developed, a particular need remained to define competencies required of professionals who practice chronic disease prevention and control. To that end, the National Association of Chronic Disease Directors (NACDD) engaged a group of stakeholders in developing competencies for chronic disease practice.

**Methods:**

Concept mapping was blended with document analysis of existing competencies in public health to develop a unique framework. Public health experts reviewed the results, providing extensive and richer understanding of the issues.

**Results:**

The final product presents an integrated picture that highlights interrelationships among the specific skills and knowledge required for leading and managing state chronic disease programs. Those competencies fall into 7 clusters: 1) lead strategically, 2) manage people, 3) manage programs and resources, 4) design and evaluate programs, 5) use public health science, 6) influence policies and systems change, and 7) build support.

**Conclusion:**

The project yielded a framework with a categorization scheme and language that reflects how chronic disease practitioners view their work, including integrating communications and cultural competency skills into relevant job functions. Influencing policies and systems change has distinct relevance to chronic disease practice. We suggest uses of the competencies in the field.

## Introduction

We describe a project of the National Association of Chronic Disease Directors (NACDD) to develop a comprehensive set of competencies for public health practitioners working in chronic disease prevention and control. The project focused on the specific knowledge and skills required in the field — what managers and leaders of chronic disease programs in states need to know and be able to do. The identification of competencies needed by public health practitioners, creation of competency-based curricula, and assurance that professionals have mastery of competencies have been emphasized as priorities by the US Public Health Service (1), the Centers for Disease Control and Prevention (2), the Institute of Medicine (3,4), and the Public Health Agency/Faculty Forum (5). Competencies specific to chronic disease practice will allow professional development to be tailored to the unique aspects of chronic disease.

The project reported here focused specifically on the way knowledge and skills are used in the *practice* of chronic disease prevention and control in public health. Managers will gain a richer understanding of the competencies required on the job in this field and can use the framework as the basis for planning their own or their staff's professional development. These competencies can be used to re-examine job descriptions, job requirements, and training needs.

We synthesized literature on competencies in public health and input from brainstorming sessions with chronic disease practitioners to identify a set of chronic disease competencies. We then used concept mapping (6-9) to organize these competencies into a framework. Concept mapping is a well-documented social research method (6-11) that has been used in public health projects (12-14).

## Methods

All new public health competencies are expected to conform to 2 documents: *Core Competencies for Public Health Professionals* (15) and *Essential Public Health Services* (16). The Council on Linkages Between Academia and Public Health Practice, funded by the Health Resources and Services Administration, used a lengthy review process involving more than 1,000 professionals to develop the *Core Competencies* (15). The 17 Council on Linkages member organizations from across the spectrum of public health endorsed *Core Competencies* (15) as a framework and incentive for competencies development.

*Essential Services* undergirds the National Public Health Performance Standards Program to improve the quality of public health and the performance of public health systems (16). Many public health professionals have applied *Essential Services* to their activities.

However, neither *Core Competencies* nor *Essential Services* provides direction to specific public health practice specialties, nor were they intended to do so. Although other public health specialties have created their own competencies, no consensus has been reached on the competencies required for chronic disease practice.

The first step was to identify and develop a possible set of competencies, seeking saturation on the range of knowledge and skills required of chronic disease practitioners. We analyzed literature and documents, received input from practitioners in the field, and conducted a group review of a draft set of ideas. To ensure that the competencies for chronic disease practice were well grounded in earlier initiatives, the team studied competencies from major health professional organizations, federal agencies, and prior NACDD efforts. The search, covering 1989 through March 2006, yielded 42 sources. Selected competencies were excluded if they were not relevant to chronic disease programs or focused on direct clinical care.

The third author (KQ) and another expert in concept mapping trained the fourth author (SS) on this process. The fourth author contributed content and context expertise to the analysis. Starting with the *Core Competencies* and the *Essential Services*, the fourth author analyzed each relevant competency verbatim from each source to identify statements that completed the following phrase: *"A specific thing that leaders and managers of chronic disease programs in states need to know or be able to do is . . .* ." The fourth author read each document, extracted each relevant competency verbatim, compared it with already extracted competencies and added it to a database if it contributed a new idea, and referenced the source. Through this document analysis, we derived 145 potentially applicable competencies.

We solicited input from members of a specially invited Competencies Planning Group, the NACDD Board of Directors, and the NACDD Professional Development Committee (N = 37) to brainstorm ideas that completed the same focus prompt: "*A specific thing that leaders and managers of chronic disease programs in states need to know or be able to do is *. . . ." Participants brainstormed anonymously on a dedicated Web site from March 27 to April 11, 2006. They could see the contributions of other participants, but they could not see the results of the document analysis. The brainstorming generated approximately 75 ideas.

We merged the derived set of competencies with the brainstormed ideas by eliminating duplicates and combining overlapping statements to create a set of 220 draft statements. At a half-day meeting, the Competencies Planning Group discussed each of the 220 proposed statements, deciding by consensus whether to include each statement in the final set or to edit or add ideas. The planning group selected single-idea statements on the basis of their uniqueness, clarity, brevity, and relevance to state chronic disease program managers and leaders. The facilitator (the third author, KQ) ensured that group decisions were based on 1 or more of those selection criteria and that all participants' views were heard. Statements that included the same keywords (eg, all draft statements that referenced *budgets*) were examined together to help identify nuances of meaning and redundant statements. To eliminate overlap and repetition of the same idea from different sources, the group's discussion focused on slight differences in wording, exploring the similarities and differences between the items and striving to select the statement that represented the core idea in the clearest and most relevant terms to chronic diseases prevention and control.

Other concept mapping projects (12-14) have used this *idea synthesis* or *statement reduction* process, which is described in detail by Kane and Trochim (7). The goal is to draw on the planning group's expertise to arrive at a comprehensive yet manageably sized set of competency statements. The review process yielded 100 discrete ideas. Of this set, 28 competencies came from the brainstormed statements, 26 from the *Core Competencies* (15), and the remainder from other sources. All adhered to public health workforce classification standards and formatting (17).

Next we asked the 37 participants to sort the 100 final statements into categories on the basis of similarity (17 of 37 [46%] responded), following these instructions:

1. Sort all the statements into categories (anywhere from 5 to 20 categories usually works well).
2. Put each statement into only 1 category.
3. Do not create a "miscellaneous" or "other" category with unrelated statements.
4. Group the statements according to how similar in meaning they are to one another. Do not sort according to importance or priority.

We used Concept Systems software version 4.0 (Concept Systems, Ithaca, New York) to aggregate and analyze the sorting data. This system applies multidimensional scaling (MDS) and hierarchical cluster analysis to integrate the sorting information and to develop a series of maps and reports. The Competencies Planning Group reviewed the analysis results at a June 2006 meeting, choosing labels for each cluster and grouping clusters into regions.

The fourth author (SS) interviewed 8 public health leaders selected by the planning group to gather feedback on the draft competencies framework. The interviewees were people who work with state chronic disease practitioners, including key strategic partners, people who have crossed over from state chronic disease programs to another sector, and stakeholders whose perspective may not have been captured through the concept mapping process. The 8 interviewees commented on the uniqueness of the domains to chronic disease, identified potential gaps, and recommended ways to use the competencies. These interviews provided a final, external review of the framework to ensure the comprehensiveness, face validity, and usefulness of the competency set. The planning group reviewed the interviewees' suggested additions and added 4 competencies, yielding a final set of 104 competencies (Appendix A). Having these experts comment on the overall framework rather than participating in the earlier brainstorming offered a fresh perspective for critique.

The NACDD Professional Development Committee discussed and reviewed all results in August 2006. No additions or revisions were proposed, suggesting both face validity and saturation of the conceptual territory.

## Results

A point map captures the underlying structure for the conceptual maps. The MDS analysis translates qualitative judgments about similarity into quantitative distances on a 2-dimensional map where each statement is represented by a numbered point. Statements closer together on the map are conceptually similar, according to participants (ie, more participants paired those ideas together into the same category in individual sorting). Statements farther apart are more dissimilar (Appendix B).

Using hierarchical cluster analysis of the MDS output, the third author (KQ) chose a 7-cluster solution that best fit the statement set, creating the point cluster map (Figure). The planning group reviewed that solution and labeled the 7 clusters: 1) use public health science, 2) design and evaluate programs, 3) manage resources, 4) manage people, 5) lead strategically, 6) build support, and 7) influence policies and systems change.

**Figure.**
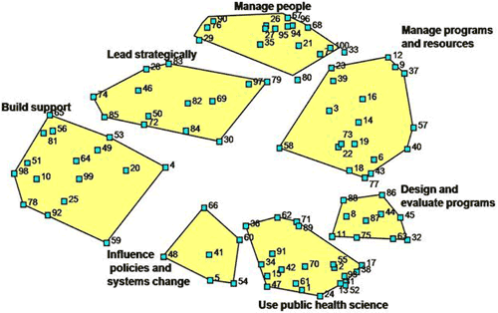
Point Cluster Map of Competencies for Chronic Disease Practice

Just as the distance relationships between the points signify degree of similarity, so do the distance relationships between clusters. For instance, a region of clusters on the left side of the map (*manage people*, *lead strategically*, *build support*, and parts of *influence policies and systems change*) are people-oriented. Those on the right side of the map (*manage resources*, *design and evaluate programs*, *use public health science*, and parts of *influence policies and systems change*) primarily involve data, systems, information, and technical aspects of the job. Planning group members also overlaid a time-management perspective onto the map. Clusters in the upper right (*manage people* and *manage resources*) occupy much of the day-to-day, immediate activities of chronic disease leaders and managers. Clusters in the lower right (*design and evaluate programs* and *use public health science*) are intermediate-term activities. Clusters on the left represent important long-term strategic objectives.

Following conventions used in the *Core Competencies* (15), the clusters became the competency domains for NACDD's Competencies for Chronic Disease Practice. Each domain represents a sphere of activity or function and contains a detailed set of specific, related competency statements (Appendix A).

NACDD Professional Development Committee members identified the following domains as being practiced most uniquely in chronic disease prevention and control (in this order):

1. Influence policies and systems change.
2. Build support.
3. Lead strategically.
4. Design and evaluate programs.

The Professional Development Committee's ranking was consistent with input from the public health leaders who were interviewed. Three of the 4 competencies added by the interviewees were in the clusters *influence policy and system changes* and *build support*.

## Discussion

A content analysis of the literature, coupled with concept mapping (6) and expert interviews, yielded a map of the skills and knowledge specific to leading and managing chronic disease programs that improve public health. The stakeholders who contributed to the organizing structure produced a collaboratively authored framework of competencies. The map aggregates each participant's conceptual sorting of the 100 ideas. By converting similarity (qualitative sorting information) into a physical location on the map, the conceptual map shows how participants viewed relationships between competencies and reflects a practice-oriented perspective.

The domains in this framework integrate knowledge and skills as they are applied to job-related activities. For instance, the Competencies for Chronic Disease Practice address communication skills where the skill applies to a job function. Many communication-related competencies are included in *build support*. That cluster focuses on communication toward the goal of establishing working relationships with stakeholders to build support for chronic disease prevention and control. Other communication skills are in the cluster *influence policies and systems change*. The focus here is on implementing strategies to change the health-related policies and systems of private or public organizations. The clusters *build support* and *influence policies and systems change* are adjacent on the map, indicating they are closely related, but distinct. Thus, the communication skills are represented on the map in relation to how they contribute to major job-related functions or tasks.

Cultural competency skills also are integrated where the skill would be most readily applied, rather than emerging as a separate cluster. For instance, the statement *apply principles of cultural appropriateness to program design* is grouped with other statements about program design in *design and evaluate programs*. The statement *recruit and retain a diverse chronic disease workforce* implies cultural competencies related to the task *manage people*. Other statements regarding cultural congruency appear in the cluster *use public health science*. Thus, skills are integrated in relation to specific job functions and tasks, illustrating an applied, practice-oriented perspective.

The project also suggests areas that may be practiced in unique ways in chronic disease. NACDD members and expert interviewees described the cluster *influence policies and systems change* as unique to chronic disease prevention and control, compared with other public health specialties. *Build support* and *lead strategically* also may be practiced in a unique way in chronic disease prevention and control. These areas are near each other on the concept map and are also the domains interpreted as part of the long-term strategic work of program directors and managers. Program design and policy and systems change — both of which are forms of intervention in public health — are divided into separate clusters in the NACDD framework, which further suggests that policy and systems change may involve a unique application of skills in the chronic disease field. Practitioners seem to perceive that the science of public health makes an important contribution to both types of interventions, based on the fact that the cluster *Use public health science* lies between these 2 types of interventions. These findings invite further exploration of how those particular domains are practiced in chronic disease prevention to ensure that professional development and educational opportunities for chronic disease practitioners address the actual requirements of this subspecialty in public health.

The Competencies for Chronic Disease Practice can be used to shape individual professional development plans. They might also form the basis for job descriptions in this public health specialty. Human Resources personnel and chronic disease leaders may use these competencies to tailor a standard template to a specific position. These competencies may prompt a re-examination of common assumptions about basic job requirements. For instance, on the basis of the framework, experience in program management may be equally or even more important to many jobs in contemporary chronic disease prevention and control than clinical certifications such as registered nurse or registered dietitian.

The Competencies for Chronic Disease Practice can also form the foundation for professional development curricula for chronic disease practitioners. Professional development providers may develop courses that address specific clusters. Because the competencies describe job-related functions, using this framework as a curricular foundation may help ensure that professional development activities are job-related and have the greatest appeal to practitioners. Cross-cutting issues like cultural competency and communication should be integrated into many courses, focusing on how skills apply to specific job activities. Finally, courses could include case studies and examples drawn from chronic disease practice, helping to ensure relevance and applicability.

Only 17 participants completed the sorting process underlying the map's organizing framework, which constitutes a limitation of this project. Map results tend to stabilize at about 25 to 30 participants (14). Thus, input from additional participants may have led to a slightly different final map configuration. Nonetheless, the map framework seems to have face validity with practitioners, as broader audiences (eg, the external interviewees and subsequent NACDD general members' presentations) have responded well to it.

Given the burden of chronic disease in this country, it is important to describe the skills and knowledge specific to leading and managing chronic disease programs. This effort describes the elements of chronic disease practice and forms the foundation for recruitment and succession planning, professional development planning, assessment and performance reviews, and curriculum development that will assure a well-educated and competent chronic disease workforce in public health.

## Appendices

### Appendix A. Competencies for Chronic Disease Practice by Domain

**Table:**

***Use Public Health Science:***

Articulate evidence-based approaches to chronic disease prevention and control (70).^a^
Discuss the underlying causes and management of chronic diseases, including behavioral, medical, genetic, environmental, and social factors (91).
Articulate key chronic disease issues (15).
Recognize and apply current relevant scientific evidence (17).
Describe socioeconomic and behavioral determinants of health disparities (42).
Develop and adapt approaches to problems that take into account differences among populations (62).
Explain relevant inferences from quantitative and qualitative data (61).
Apply ethical principles to the collection, maintenance, use, and dissemination of data and information (36).
Discuss quantitative evaluation (93).
Identify relevant and appropriate data and information sources for chronic disease (52).
Monitor and analyze chronic disease epidemiology and surveillance data to identify burden, trends, and outcomes (38).
Identify the factors that influence the delivery and use of public health programs and services (34).
Guide the translation of research into chronic disease programs and activities (89).
Know and apply the Chronic Disease Indicators (55).
Select and use appropriate data collection methods (24).
Discuss issues of data integrity and comparability (1).
Explain basic clinical terms and etiology for chronic diseases (2).
Discuss qualitative evaluation (31).
Define and interpret nontraditional data to address chronic disease prevention and control (eg, transportation data, cigarette sales) (13).
Implement social marketing strategies (71).
Maintain up-to-date knowledge on the development of genetic advances and technologies relevant to chronic diseases (47).

***Design and Evaluate Programs:***

Use program evaluation findings to improve program performance (63).
Select appropriate program and intervention activities (86).
Identify and use public health data as a tool to develop and prioritize community-based interventions or policies for chronic disease (75).
Apply principles of cultural appropriateness to program design (88).
Develop evaluation plans for chronic disease programs and activities (32).
Know program-specific content areas (45).
Apply cost-effectiveness, cost-benefit, and cost-utility analyses as appropriate (44).
Identify a data analysis agenda for state chronic disease programs (11).
Create and interpret logic models for chronic disease programs (8).
Guide the application of basic research methods and theories used in chronic disease prevention and control (87).

***Manage Programs and Resources:***

Manage chronic disease programs within budget constraints (12).
Navigate cooperative agreements with the CDC (23).
Set program goals and objectives of chronic disease programs (43).
Monitor chronic disease program performance (40).
Identify and assess potential funding opportunities (14).
Balance needs, requirements, partnerships, workload, etc, for multiple projects/programs (16).
Adhere to public health laws, regulations, and policies related to chronic disease prevention and control (58).
Prepare proposals for funding from a variety of sources (57).
Implement strategies for transition from planning to implementation (77).
Provide technical assistance to partners, subcontractors, and others as needed (22).
Develop and justify a line-item budget (37).
Assess an organization's implementation readiness, capacity, and effectiveness (73).
Conduct internal and external needs and assets assessments to inform program planning (19).
Develop and justify an activity-based budget (9).
Apply current techniques in decision analysis and planning for chronic disease (6).
Conduct regular and purposeful site visits with grantees (39).
Apply organizational theory to professional practice (3)
Develop a plan for chronic disease information systems (18).

***Manage People:***

Manage a team of professional staff effectively (96).
Balance multiple tasks (100).
Prioritize work responsibilities of self and staff (94).
Practice effective time management (7).
Recruit and retain a diverse chronic disease workforce (27).
Implement processes so that staff from multiple programs can identify underlying common goals and outcomes (80).
Match staff skills to tasks (68).
Recruit, mentor, and support a diverse interdisciplinary team (95).
Mediate and resolve conflicts effectively (29).
Conduct performance appraisals and give guidance/feedback to staff regularly (33).
Promote team and organizational learning (90).
Support professional and personal development for chronic disease program staff (76).
Negotiate budgets and contract requirements/objectives with both funders and contractors (26).
Navigate relevant fiscal systems effectively (67).
Manage meetings and conferences (21).
Employ effective interviewing and questioning strategies (35).
Motivate individuals and teams to achieve goals.^b^
Facilitate integration between chronic disease programs and other state health-related programs (eg, surveillance, oral health, maternal and child health, Medicaid, state employee health insurance, emergency service providers and planners) (20).
Use effective collaboration strategies to build meaningful partnerships (64).
Lead and participate in groups to address emerging chronic disease issues (99).
Prepare and present the business case for chronic disease prevention effectively (4).
Facilitate use of coalitions as effective change agents for chronic disease prevention and control (10).
Develop enough social capital and political savvy to navigate the appropriate organizational systems quickly (53).
Facilitate group interactions and decision making (49).
Work collaboratively with partners on data collection and interpretation (59).
Use the media, advanced technologies, and community networks to communicate information (92).
Identify and describe the roles of the key players on a national level (56).
Participate in national work groups to facilitate effective implementation of chronic disease programs (51).

***Lead Strategically:***

Demonstrate critical thinking (84).
Respond with flexibility to changing needs (97).
Leverage resources (83).
Provide leadership to create key values and shared vision (28).
Generate, share, and accept new ideas and incorporate them (85).
Apply effective problem-solving processes and methods (79).
Facilitate integration among chronic disease programs (72).
Create a culture of ethical standards within organizations and communities (74).
Develop budget initiatives based on priorities to sell to decision-makers (46).
Oversee the development and implementation of a statewide chronic disease plan (30).
Translate policy into organizational plans, structures, and programs (69).
Identify policy agenda for state chronic disease programs (50).
Identify individual and organizational responsibilities within the context of the Essential Public Health Services and core functions (82).

***Build Support:***

Establish and maintain linkages and/or partnerships with key stakeholders (including traditional, nontraditional, and academic partners) (25).
Interact effectively with other major sectors (including the health care industry, transportation, parks and recreation, education, and the private sector).^b^
Communicate effectively in writing for professional and lay audiences (78).
Listen to others in an unbiased manner, respect points of view of others, and promote the expression of diverse opinions and perspectives (65).
Communicate effectively orally for professional and lay audiences (98).
Advocate for chronic disease programs and resources (81).

***Influence Policies and Systems Change:***

Explain systems thinking and principles of systems change (41).
Use policy as a tool in advancing chronic disease and control (66).
Present accurate demographic, statistical, programmatic, and scientific information effectively for professional and lay audiences (48).
Assess the impact of public policies, laws, and regulations on chronic disease prevention and control (60).
Influence policy through accurate, persuasive communications with the public, partners, health agency leaders, and policy makers.^b^
Articulate relative risks of disease effectively (5).
Describe the historical development, structure, and interaction of public health and health care systems (54).
Use health economics concepts and language to present chronic disease programs in a convincing manner to appropriate audiences.^b^

*Source:* National Association of Chronic Disease Directors, 2006. Used with permission.

a The numbers in parentheses are randomly assigned identifiers that enable the reader to cross-reference the statement with its location on the concept map in the Figure. Statements without a number were added via subsequent discussions with interviewees.

b Item added at the suggestion of interviewees.

### Appendix B. Discussion of Reliability of Concept Mapping Results for the Competencies for Chronic Disease Practice Map Analysis

Further details of the concept mapping analysis can be found in Kane and Trochim, 2007 (7). Stress value (10,18) is typically reported for concept mapping projects as a measure of reliability. In this project, the stress value (a measure of reliability) for the multidimensional scaling (MDS) results was 0.25. Low stress values mean greater correspondence between the similarity matrix (the aggregation of all participants' sorts and the MDS input) and the distance map (the MDS output). General guidelines suggest that stress values between .05 and .35 are acceptable, depending on the stage and purpose of the research (10,18,19). In a study of the reliability of concept mapping, Trochim (10) reported that the average stress value across 33 projects was 0.29 with a range of 0.16 to 0.35. This project's stress value was lower (ie, better) than average.

## References

[1] (1997). US Public Health Service. The public health workforce: an agenda for the 21st century.

[2] Centers for Disease Control and Prevention Proceedings from the Public Health Workforce Expert Panel Workshop.

[3] Institute of Medicine (1988). The future of public health.

[4] Institute of Medicine (2003). The future of the public's health.

[5] Sorenson AA, Bialek RG (1991). The Public Health Faculty/Agency Forum: linking graduate education and practice: final report submitted to the Bureau of Health Professions, Health Resources and Services Administration (US) and Centers for Disease Control and Prevention (US).

[6] Trochim W (1989). An introduction to concept mapping for planning and evaluation. Eval Program Plann.

[7] Kane M, Trochim WMK (2007). Concept mapping for planning and evaluation.

[8] Trochim W (1989). Concept mapping: soft science or hard art?. Eval Program Plann.

[9] Trochim W, Kane M (2005). Concept mapping: an introduction to structured conceptualization in health care. Int J Qual Health Care.

[10] Trochim W Reliability of concept mapping. Paper presented at: Annual Conference of the American Evaluation Association.

[11] Burke JG, O'Campo P, Peak GL, Gielen AC, McDonnell KA, Trochim W (2005). An introduction to concept mapping as a participatory public health research methodology. Qual Health Res.

[12] Trochim W, Milstein B, Wood BJ, Jackson S, Pressler V (2004). Setting objectives for community and systems change: an application of concept mapping for planning a statewide health improvement initiative. Health Promot Pract.

[13] Wheeler FC, Anderson LA, Boddie-Willis C, Price PH, Kane M (2005). The role of state public health agencies in addressing less prevalent chronic conditions. Prev Chronic Dis.

[14] Anderson LA, Gwaltney MK, Sundra DL, Brownson RC, Kane M, Cross AW (2006). Using concept mapping to develop a logic model for the Prevention Research Centers Program. Prev Chronic Dis.

[15] (2001). Council on Linkages Between Academia and Public Health Practice. Core competencies for public health professionals.

[16] (1994). Core Public Health Functions Steering Committee. Essential public health services. Centers for Disease Control and Prevention. National Public Health Performance Standards Program.

[17] Gebbie K (2004). Competency-to-curriculum toolkit: developing curricula for public health workers.

[18] Bartholomew DJ, Steele F, Moustaki I, Galbraith JI (2002). The analysis and interpretation of multivariate data for social scientists.

[19] Kruskal JB, Wish M (1978). Multidimensional scaling.

